# Dairy production in an urbanizing environment—Typology and linkages in the megacity of Bengaluru, India

**DOI:** 10.1371/journal.pone.0255791

**Published:** 2021-08-12

**Authors:** Marion Reichenbach, Ana Pinto, Sven König, Raghavendra Bhatta, Eva Schlecht

**Affiliations:** 1 Animal Husbandry in the Tropics and Subtropics, University of Kassel and Georg-August-Universität Göttingen, Witzenhausen, Germany; 2 Animal Breeding and Genetics, Justus-Liebig-Universität Gieβen, Gieβen, Germany; 3 ICAR National Institute of Animal Nutrition and Physiology (NIANP), Bengaluru, India; National Veterinary School of Toulouse, FRANCE

## Abstract

Urbanization is a main driver of agricultural transition in the Global South but how it shapes trends of intensification or extensification is not yet well understood. The Indian megacity of Bengaluru combines rapid urbanization with a high demand for dairy products, which is partly supplied by urban and peri-urban dairy producers. To study the impacts of urbanization on dairy production and to identify key features of dairy production systems across Bengaluru’s rural-urban interface, 337 dairy producers were surveyed on the socio-economic profile of their household, their dairy herd and management, resources availability and, in- and output markets. A two-step cluster analysis identified four spatially explicit dairy production systems based on urbanization level of their neighborhood, reliance on self-cultivated forages, pasture use, cattle in- and outflow and share of specialized dairy genotypes. The most extensive dairy production system, common to the whole rural-urban interface, utilized publicly available feed resources and pasture grounds rather than to cultivate forages. In rural areas, two semi-intensive and one intensive dairy production systems relying on self-cultivation of forage with or without pasture further distinguished themselves by their herd and breeding management. In rural areas, the village’s dairy cooperative, which also provided access to inputs such as exotic genotype through artificial insemination, concentrate feeds and health care, was often the only marketing channel available to dairy producers, irrespective of the dairy production system to which they belonged. In urban areas, milk was mostly sold through direct marketing or a middleman. Despite rapidly progressing urbanization and a population of 10 million, Bengaluru’s dairy sector still relies on small-scale family dairy farms. Shifts in resources availability, such as land and labor, are potential drivers of market-oriented intensification but also extensification of dairy production in an urbanizing environment.

## Introduction

As rural areas are taken over by rapidly expanding cities, the spatial flow of agricultural products between rural producers and their urban consumers is at the heart of complex social-ecological systems (**SES**) extracting local resources [1–3]. Producers and consumers within a same SES are further linked by different flows of material, people, information and financial capital: on one hand, through consumption patterns, urban consumers influence the goods and services produced by farmers and their management practices [4]. It thus shapes farmers’ use of critical agricultural resources (land, water, capital and labor) and accordingly, agricultural production systems. On the other hand, environmental externalities of the thus-shaped agricultural production systems act as a feedback to urban consumers [4]. When maintaining an equilibrium between local resource use and urban population, SES are depicted as “green-loop” [3–5]. By its nature and scope, urbanization however increases the risk of breaking off this equilibrium: one impact of urbanization is the dichotomization of rural and urban worlds spatially and on a sectoral basis, with the rural space dedicated to agricultural production and the urban one to consumption [1, 6]. This dichotomization leads to further distance between producers and consumers: i) a psychological one; that is the concerns of the consumers regarding social and ecological consequences of their consumption decrease, especially regarding negative environmental externalities [2]; and ii) a structural one; that is intermediaries in the value chain are multiplied as durability of primary agricultural products is increased by processing, and as transport distances increase [4, 7, 8]. Urbanization also nurtures homogenization of production systems and products, which guarantees to urban consumers the quality and safety of products [4, 9, 10]. A further impact of urbanization is agricultural intensification due to i) decreased availability of land, because of conversion of agricultural land into built-up areas and the fragmentation of the agricultural landscape, and labor, as people, especially young, move to cities in search of better economic opportunities [1, 11]; ii) increased farmers’ access to inputs and marketing channels [12]. The urbanization level of an environment thus represents a distinct set of opportunities and constraints for farmers in terms of available resources and resource flows connecting social-ecological components, shaping a variety of production systems. A system approach is then crucial to understand the impacts of urbanization on resource use and linkages’ quality of the SES, in which farmers are embedded. Urbanization is now the most intense in Asia and Africa [13]. In various major West African cities [14–16], distinct livestock production systems coexisting within the same urban and peri-urban space have been documented, ignoring however the livestock production systems at the rural periphery of the cities and the SES in which they are embedded.

Being one of the fastest urbanizing countries [17], India hosts the second largest population in the world (1.37 billion in 2019) [18] with presently 34% of its population living in cities [19]. With 29% of the population being vegetarian [20], milk is a vital source of animal protein. In the 1970s’, the Indian government launched a decades-long development program called *Operation Flood* that focused on dairy production as a vital rural-urban linkage [21]. Operation Flood successfully scaled up rural milk production, marketing and processing through dairy cooperatives and improved infrastructures, to supply urban areas [21]. Thus, taking dairy production in Bengaluru, India, as an example for production systems in an urbanizing environment and a discussion point on social-ecological linkages in the same context, the present study considers urban, peri-urban and rural areas in and around an emerging megacity to tackle the following research questions: do distinct dairy production systems (**DPS**) coexist along a rural-urban interface and how are they impacted by urbanization? To answer these questions, we surveyed 337 dairy producers in and around the emerging megacity of Bengaluru in southern India.

## Materials and methods

### Research area

**Fig 1:**
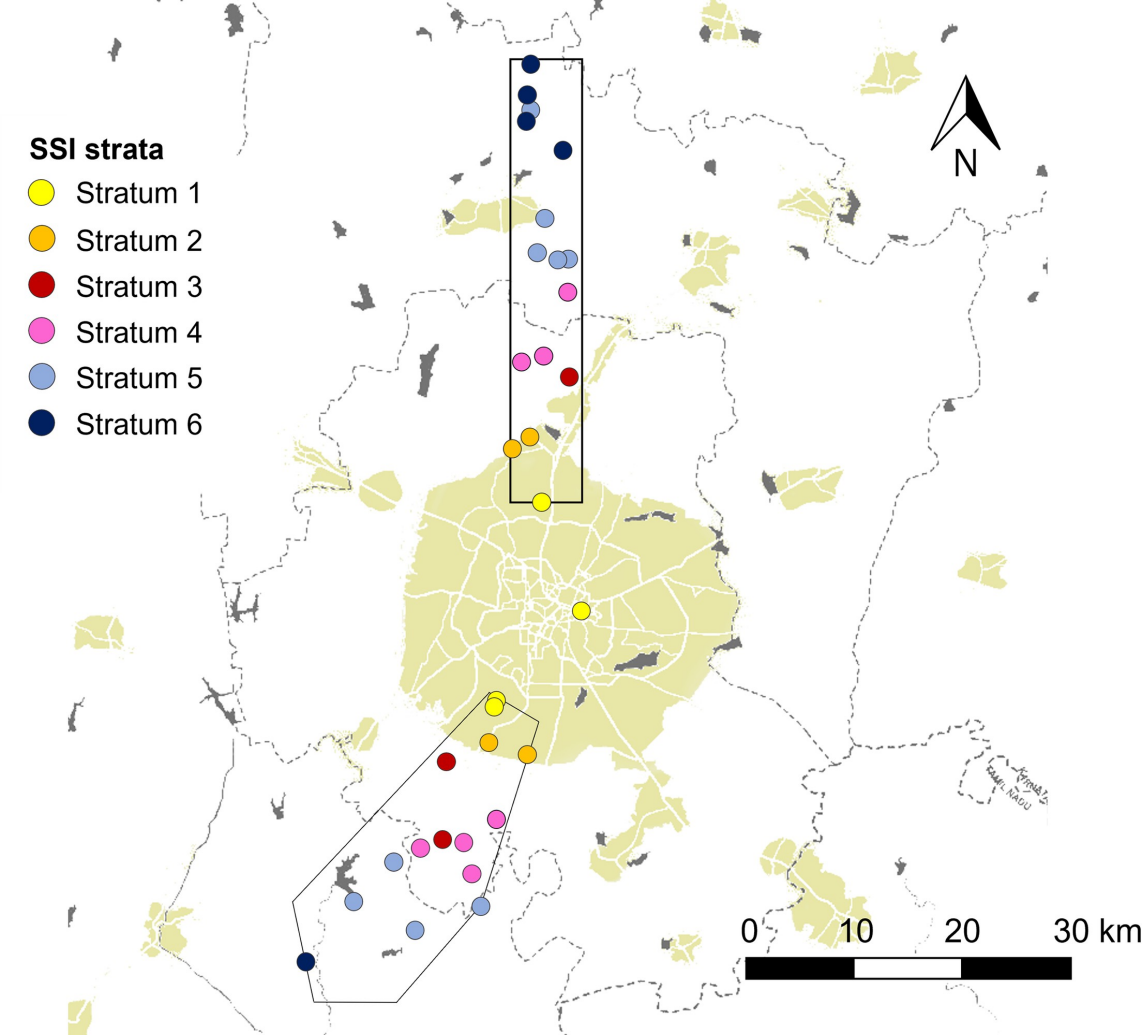
Map of Bengaluru (built-up area in color), northern and southern research transects and selected settlements (dots) per stratum (colors) of the Survey Stratification Index (SSI) with 1 = urban and 6 = rural.

Capital of the southern Indian state of Karnataka, Bengaluru has a hot semi-arid climate (average 2013–2017: monthly maximum temperature 29.5°C, monthly minimum temperature 18.5°C, 948 mm of annual rainfall—Weather station data of the University of Agricultural Sciences Bengaluru). The dry season (March-May) is followed by a monsoon season (June-October) and winter (November-February). Bengaluru’s urban agglomeration is amongst the largest in India, driven since the 1970s by an unprecedented growth of population, which is now more than 10 million [22, 23]. The State of Karnataka inaugurated its dairy development program as early as 1974, based on the model of Operation Flood, setting up the Karnataka Milk Federation (known as *KMF*) [24]. Two research transects were established within Bengaluru’s rural-urban interface, following an urban to rural gradient: the northern transect was a rectangular stripe of 5 km width and 40 km length along a north-south axis, starting 10 km away from the city center, in the northern part of Bengaluru (Fig 1). The southern transect was a ca. 300 km^2^ polygon along a south-west axis of 30 km length, starting 10 km away from the city center, in the southern part of Bengaluru. Each settlement (village, suburb or urban neighborhood) within the two transects was identified and assigned a survey stratification index (**SSI**) stratum. The SSI went from stratum 1 = urban to 6 = rural, based on build-up density of the settlement and its distance to Bengaluru’s center as proxy for its urbanization level [25]. Urbanization levels were “urban” (strata 1 and 2), “peri-urban” (strata 3 and 4) and “rural” (strata 5 and 6) [25].

### Sampling design

A two-step random selection process was used to survey a minimum of 300 dairy producers across both transects: 30 settlements, 15 in the northern transect and 15 in the southern one (S1 Table), were first drawn at random proportionally to the transects’ prevalence of settlements per SSI stratum [25]. In a second step, dairy producers were randomly selected per settlement, based on the latest vaccination list for foot-and-mouth disease (mandatory vaccination campaign done every 6 months). In two urban settlements, the vaccination list was not available. Thus, the total number of dairy producers was assessed by scouting the settlement on foot and talking to local inhabitants. As the southern transect was more urbanized than the northern one [25] and to compensate for the lower number of dairy producers in urban settlements (9 ± 7 dairy producers) than in peri-urban (45 ± 37) and rural ones (55 ± 26; correlation coefficient SSI: total number of dairy producers per settlement = 0.55, P < 0.05), i) the selection threshold of dairy producers per settlement was set at 20% in the northern transect and at 30% in the southern one; ii) two urban settlements were purposefully added, one in the northern transect (stratum 2, selection threshold = 20%, 2 surveys), and one close to the city center, thus mid-way between the two transects (stratum 1, selection threshold = 30%, 4 surveys). To assure potentially continuous, even though minimal, involvement in milk marketing, only dairy producers with two or more dairy cattle considered as productive assets were surveyed. Based on first insights from the field, dairy cattle considered as productive assets and further referred to as **LDH** were: lactating (**L**) or dry (**D**) cows, plus mature heifers (**H**; pregnant or inseminated at least once), which were cared for in a similar way as cows, although not productive *per se*. *Bos taurus* Holstein Friesian and Jersey were considered as “exotic” genotypes, as opposed to “native” *Bos indicus* genotypes–mostly Hallikar, an indigenous draught breed from the State of Karnataka with low milk production potential (2.4 kg per day) [26]. The lack of breeding records prevented the distinction between different types of crossbreeds, despite dairy producers identifying specific (multigeneration) crossbreeds as “All-Black”or “Half-Black”. A total of 337 dairy producers were surveyed between mid-August and mid-November 2016 (59% in rural settlements, 33% in peri-urban and 8% in urban ones).

### Dairy production baseline survey

A detailed protocol of the sampling process, the information sheet for participants and the survey in itself were submitted for critical review and approval to both the University of Agricultural Sciences Bangalore and the ICAR National Institute of Animal Nutrition and Physiology in Bengaluru; both research institutes are collaborating closely with local farmers through research and extensions services. Especially survey-based research is often done at the University of Agricultural Sciences Bangalore and thus, the institute is known in Bengaluru’s rural-urban interface and benefits from the farmers’ trust. Our research involved solely survey-based data from adult participants and was considered as including no critical questions by both research institutes. As such, after standardization of procedures during a pre-testing phase and feedback from about 10 households to the survey’s enumerators and researchers, the survey was approved by both research institutes. All surveys were conducted in Kannada, the official language of the State of Karnataka, face-to-face with the dairy producer himself/herself or an adult member of his/her household. Before starting the survey, the purpose and scope of the survey was explained and only respondents giving oral consent to participate were surveyed. Every survey was conducted by a team of two persons: one translator, familiar with the questions and one researcher, filling out the survey sheets while checking for plausibility and consistency of answers. The survey lasted for 28 minutes on average. Collected quantitative and qualitative data addressed the socio-economic profile of the dairy producer, dairy herd composition and management with focus on breeding, health care and feeding, in- and output markets for dairy production and further agricultural activities following previous system topologies [16, 27]. Several household characteristics were calculated following established standards: this applied to the socio-economic classification of the households according to the Market Research Society of India’s system [28], calculation of household labor force [29] and of tropical livestock units [15]. Data were treated anonymously but the location of each dairy farm was georeferenced with a wireless GPS logger (Holux M-241), with priority given to the location of the cowshed if separated from the house of the dairy producer.

### Statistical analyses

Surveyed dairy producers within Bengaluru’s rural-urban interface were grouped through a two-step cluster analysis (IBM SPSS Statistics 20) as it can simultaneously handle quantitative and qualitative variables. Following previous system topologies [16, 27], quantitative and qualitative variables relevant for dairy production according to expert knowledge were first selected, based on completeness, consistency and (frequency) distribution of the answers. Strongly correlated variables were excluded (P < 0.01, Pearson correlation coefficient > 0.7), resulting in 26 main independent variables selected. A pre-screening of the data through categorical principal component analysis excluded five variables accounting for little variability (loading score < 0.5 on components with eigenvalue > 1). Several clustering runs were explored with the remaining 21 variables. The number of clusters was restricted to 3–5 to avoid low and unbalanced numbers of dairy producers per cluster and allow for meaningful interpretation of the cluster solution as a base for further investigations. A four-cluster solution, based on 5 coherent predictors and a fair silhouette measure of cohesion (0.3), was finally chosen. Collected data were then analyzed per DPS to understand the trends of agricultural transition within an urbanizing environment. In addition to descriptive statistics depicting arithmetic mean and standard deviation (±) for relevant variables, chi-squared and Kruskal-Wallis tests were used on non-clustering variables to describe each DPS. Post hoc tests used were Pearson residuals (threshold at ± 1.96) or pairwise Wilcoxon rank sum tests (Holm correction for pairwise comparison). Significance was declared at P < 0.05.

## Results

### Predictors of dairy production systems

**Fig 2:**
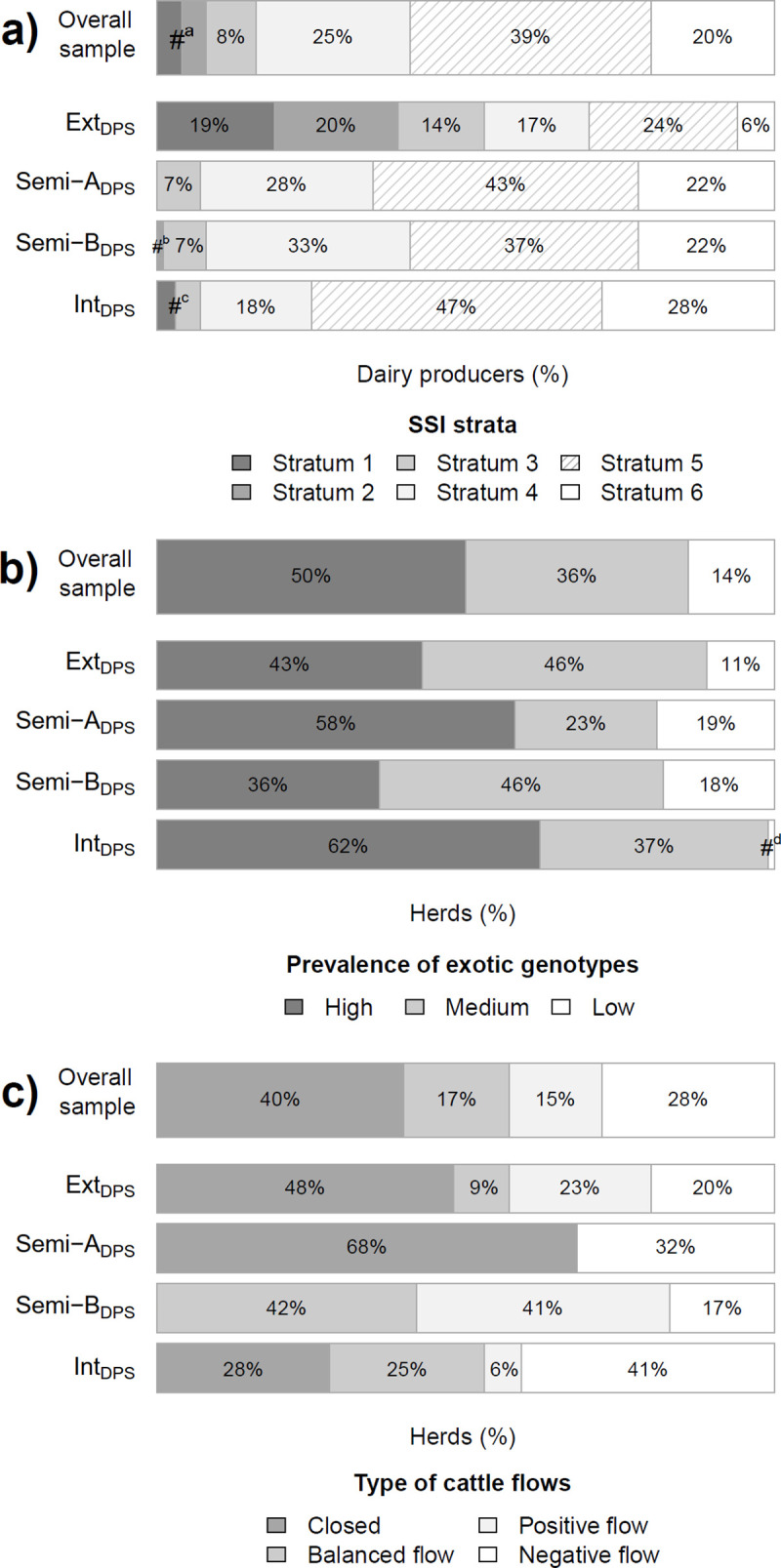
Frequency of a) dairy producers (%) according to the urbanization level of the dairy farm’s surroundings (P SSI; stratum 1 = urban to stratum 6 = rural), b) herds (%) according to the prevalence of exotic genotypes within the herd (P-GEN) and c) herds (%) according to the type of cattle flows within the herd (P-FLOW), overall and for each dairy production system (DPS). #^a^ = 4% each for stratum 1 and 2 in the overall sample; #^b^ = 1% for Semi-B_DPS_ stratum 2; #^c^ = 3% for Int_DPS_ stratum 1 and 4% for Int_DPS_ stratum 3; #^d^ = 1% for Int_DPS_, low prevalence.

**Fig 3:**
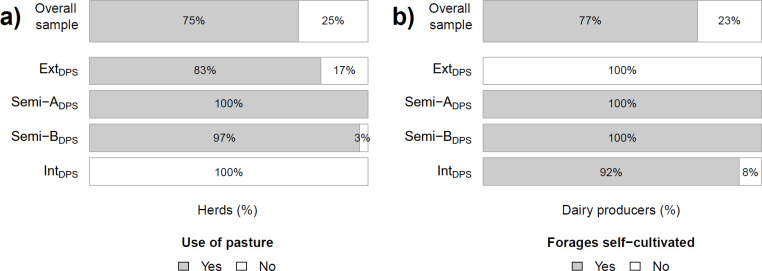
Frequency of a) herds (%) according to their use of pasture through grazing (P-PAS) and b) dairy producers according to their reliance, at least partial, on self-cultivated forages (P-FOR), overall and for each dairy production system (DPS).

The first out of the five predictors of DPS was the settlement’s SSI (**P-SSI**; predictor importance = 0.26) as proxy for the urbanization level of the dairy farm’s surroundings. The second and third predictors related to breeding/herd management: **P-GEN** captured the prevalence of exotic genotypes in the entire dairy herd (LDH plus calves and immature heifers; predictor importance = 0.07). The ownership of exotic genotypes attested a higher specialization in dairy production because of the higher financial investment needed to acquire and maintain high yielding cattle. A herd including exclusively exotic genotypes was thus given the specialization grade “high” (50% of all herds). A herd including crossbreeds and eventually exotic genotypes was given the specialization grade “medium” (36%). A herd including native genotypes and eventually crossbreeds or exotic genotypes or both was given the grade “low” (14%; Fig 2). **P-FLOW** captured the buying (inflow) and selling (outflow) of cattle within the herd during the 12-month period preceding the survey (predictor importance = 0.51). P-FLOW categorized each herd independently of the net flow and type of cattle, as follows: a herd with no cattle in- or outflow was classified “closed” (40% of all herds), a herd with both cattle in- and outflow had a “balanced flow” (17%), a herd with only inflow had a “positive flow” (15%) and a herd with only outflow had a “negative flow” (28%; Fig 2). The fourth and fifth predictors related to the feeding management (Fig 3). **P-PAS** captured the use of pasture through grazing (predictor importance = 0.90): “no” meant an absolute “absence of pasture” (25% of all herds), while “yes” meant “use of pasture” (75%), independently of the regularity and length of daily pasturing. **P-FOR** captured the reliance, at least partial, on self-cultivated forages (predictor importance = 1.00): “no” meant that the dairy producer was not cultivating any forage (23% of all herds), while “yes” meant reliance on self-cultivated forages (green or dry; 77%). Thereby no distinction was made between complete or partial reliance because i) the level of reliance on self-cultivated forage varied with season and dairy cattle as did overall diet composition, ii) the origins of a given forage type could be multiple, and iii) crop use could be multiple.

### Typology of dairy production systems

#### Ext_DPS_: Extensive and ubiquitous

**Ext**_**DPS**_ included 70 dairy producers (21%) from across the whole rural-urban interface: 39% were urban, accounting for 27 out of the 30 urban dairy producers surveyed overall (P < 0.5), 31% were peri-urban and 30% were rural (Fig 2). The predictors of breeding/herd management showed that the majority of dairy producers in Ext_DPS_ kept non-specialized herds with both exotic genotypes and crossbreeds (46%; P < 0.5), and did not usually sell or buy cattle (48%; P < 0.5; Fig 2). The feeding management of Ext_DPS_ relied on the use of pasture (P < 0.05) but not on self-cultivated forages (P < 0.05). Ext_DPS_ was thus characterized as an extensive ubiquitous DPS.

#### Semi-A_DPS_: Semi-intensive and rural, variant A

Being the largest amongst the four clusters, **Semi-A**_**DPS**_ included 120 dairy producers (35%) but none from an urban settlement (P < 0.05; Fig 2). The predictors of breeding/herd management showed that the majority of dairy producer in Semi-A_DPS_ kept exclusively exotic genotypes (58%; P < 0.05) and did not usually sell or buy cattle (68%; P < 0.05; Fig 2). The feeding management of Semi-A_DPS_ incorporated both the use of pasture (P < 0.05) and the reliance, at least partially, on self-cultivated forages (P < 0.05). Semi-A_DPS_ was thus characterized as the variant A of a semi-intensive rural DPS with variant A meaning “a closed, specialized herd”.

**Semi-B**_**DPS**_**: Semi-intensive and rural, variant B. Semi-B**_**DPS**_ included 76 dairy producers (23%) of which 59% were rural (Fig 2). In opposition to the variant A of a semi-intensive rural DPS, only 36% of dairy producers of the variant B kept exclusively exotic genotypes. The predictors of breeding/herd management showed that the majority of dairy producers in Semi-B_DPS_ kept non-specialized herds with both exotic genotypes and crossbreeds (46%; P < 0.05) and did sell (17%) or buy (42%; P < 0.05) cattle, or both (41%; P < 0.05; Fig 2). However, dairy producers of the variant B had the same semi-intensive feeding management as dairy producers of the variant A: they also made use of pasture (P < 0.5) and relied, at least partially, on self-cultivated forages (P < 0.05). Semi-B_DPS_ was thus characterized as the variant B of a semi-intensive rural DPS with variant B meaning “an open, non-specialized herd”.

#### Int_DPS_: Intensive and rural with specialized herds

**Int**_**DPS**_ included 71 dairy producers (21%) of which 75% were rural (Fig 2). The predictors of breeding/herd management showed that Int_DPS_ had the largest share of dairy producers keeping only exotic genotypes (62%, and only 1% showing low specialization of the herd; P < 0.05) and the largest share of dairy producers who sold cattle (41%; P < 0.05; Fig 2). The feeding management of Int_DPS_ was the only one not relying on pasture (P < 0.05) but only, at least partially, on self-cultivated forages (P < 0.05). Int_DPS_ was thus characterized as an intensive rural DPS with specialized herds.

### Key features of dairy productions systems

#### Additional spatial patterns

Despite P-SSI accounting for location within Bengaluru’s rural-urban interface, there were additional system-specific spatial patterns: at the transects’ scale, Int_DPS_ was more common in the northern transect than in the southern one (P < 0.05). At the settlements’ scale, dairy producers of a same settlement were often regrouped in the same DPS (P < 0.05) even in rural areas where more than one DPS existed; this shows that conditions at settlement level, such as climate, feed resource availability or marketing opportunities, impacted DPS.

#### Socio-economic profile of dairy producers

The socio-economic profile of Bengaluru’s dairy producers was homogenous across the four DPS and provided a clear picture of the local dairy sector: in Bengaluru, dairy production was a *family business*, with the household head typically being a married man, 53 ± 13 years old, with not more than the mandatory school education but 22 ± 14 years of experience in dairy production, whose parents also had owned cattle (79% of household heads). He was chief earner of the household (74%), which included 4 ± 2 additional members, often spread across three generations. Labor force of the household amounted to 3.6 ± 1.6. Including the household head, 3 ± 2 household members were involved in dairy production, but their amount of work varied. Only 4% of the dairy producers hired extra labor, corroborated by a low importance ranking (0 ± 0.2) of labor as expenses related to dairy production (Table 1). In the household of 53% dairy producers, at least one household member (1.4 ± 0.6; in 66% of the cases a member of the younger generation) was involved in an off-farm economic activity. Based on their socio-economic profile, 66% of the households were classified as “Indian middle class*”*, whereby the importance of dairy production as an income source differed among DPS: with the exception of Ext_DPS_, most dairy producers had mixed income sources with dairy production as the major source of income (48% of dairy producers in Semi-A_DPS_, 45% in Semi-B_DPS_ and 42% in Int_DPS_) or a complementary one (29% of dairy producers in Semi-A_DPS_, 38% in Semi-B_DPS_ and 35% in Int_DPS_). In Ext_DPS_, the majority of dairy producers had only dairy production as their source of income (36%; P < 0.05). It is also worth mentioning that in Semi-A_DPS_, dairy production was not an important income source for 12% of dairy producers (P < 0.05).

**Table 1:** Importance ranking scores of expenses related to dairy production with 3 = first expense, 2 = second expense, 1 = third expense and 0 = not an expense.

DPS	Importance ranking score					
	Concentrate feeds	Forages	Health care	Reproduction	Land	Labor
Ext_DPS_	2.8 ± 0.6	1.5 ± 1.1^a^	0.5 ± 0.8	0.1 ± 0.5*	0	0
Semi-A_DPS_	2.7 ± 0.9	0.8 ± 1.1^b^	0.7 ± 0.9	0.2 ± 0.6*	0 ± 0.3	0
Semi-B_DPS_	2.7 ± 0.9	0.7 ± 1.0^b^	0.7 ± 1.0	0.1 ± 0.4*	0 ± 0.1	0
Int_DPS_	2.9 ± 0.5	0.8 ± 1.0^b^	0.5 ± 0.8	0 ± 0.1*	0 ± 0.2	0 ± 0.2

Values within a column with different superscript letters differs significantly (P < 0.05).

*Significant analysis of variance but small data set prohibits pairwise comparison.

#### Dairy herd

With minor variations across the four DPS, insights on dairy herd further completed the overview of Bengaluru’s dairy sector as a *small-scale* family business: the average LDH number was 3 ± 2 with 1 ± 1 additional calves and/or immature heifers kept for herd renewal. Large herds were rare with only 4 dairy producers in the whole sample owning 10 LDH or more. The average lactation number differed between the DPS: in Semi-B_DPS_ with its open breeding/herd management of cattle in- and outflow, the average lactation number was 2.1 ± 1.0, as compared to 2.6 ± 1.1 in Semi-A_DPS_ (P < 0.05), where no selling and/or buying of cattle took place and cattle were thus kept longer. Cattle in Ext_DPS_ and Int_DPS_ had an identical intermediate lactation number (2.3 ± 0.9).

#### Breeding, reproduction and health care

As captured by P-GEN, exotic genotypes were standard in Bengaluru’s rural-urban interface across all DPS, with overall more than one dairy cattle out of two being Holstein Friesian (54% of all dairy cattle) and at least one out of six being Jersey (15%). Despite the advantage of selling male calves for draught purpose, native cattle were the least common (10%); they were kept for both milk production and draught purpose (46%) or exclusively for milk production (54%). Crossbreeds (21%), from first-generation *Holstein Friesian x Jersey* or *exotic x native* to multigeneration indiscriminate crossbreeds, resulted from local breeding practices rather than being a real choice: artificial insemination was made widely available by the Karnataka Milk Federation and across the four DPS, 86% of all dairy producers relied exclusively on artificial insemination and 9% on both artificial insemination and natural mating if their first choice method failed or according to the cattle genotype. The usage was to inseminate heifers with Jersey semen, irrespective of their own genotype, to facilitate their first calving, which explained numerous *Holstein Friesian x Jersey* crossbreeds. The success of artificial insemination, however, varied amongst the DPS: only 18% of dairy producers in Semi-B_DPS_ stated that the first artificial insemination was always successful, by opposition to 35% in Ext_DPS_, 30% in Semi-A_DPS_ and 42% in Int_DPS_ (P < 0.05). Most dairy producers did not rely on natural mating due to lack of breeding bulls, especially of exotic genotypes, which further explained reliance on artificial insemination and *exotic x native* crossbreeding. Dairy producers in Int_DPS_ were quite successful in renewing their herd, with only 19% of them stating that it was harder to get a heifer pregnant than a cow (*versus* 44% in Ext_DPS_, 35% in Semi-A_DPS_ and 38% in Semi-B_DPS_; P < 0.05) and getting them inseminated at 21 ± 8 months old (*versus* 27 ± 9 in Ext_DPS_ and 27 ± 11 in Semi-A_DPS_; P < 0.05; 24 ± 10 in Semi-B_DPS_). Despite (repeated) use of artificial insemination, dairy producers did not consider reproduction costs among their three main expenses (importance ranking = 0.1 ± 0.5; Table 1) as they benefited from artificial insemination through their dairy cooperative at a low price.

Occurrence of health problems was also unrelated to DPS: one dairy producer out of four reported mastitis in his herd during the last 12 months, even though the cow’s udder was washed before milking on all farms. Moreover, 35% of the dairy producers reported additional health issues such as fever (50% of additional health issues), foot-and-mouth disease (12%, despite vaccination campaigns every 6 months), physical wounds (11%) and fertility or calving issues or both (11%). Hoof care was uncommon (practiced by only 5% of all dairy producers) as was the use of bedding material (rubber mats) in the cowshed (7%). Because of the high costs engendered by even a single health problem, dairy producers considered health care among their three main expenses (importance ranking = 0.6 ± 0.9; Table 1).

#### Nutrition

**Fig 4:**
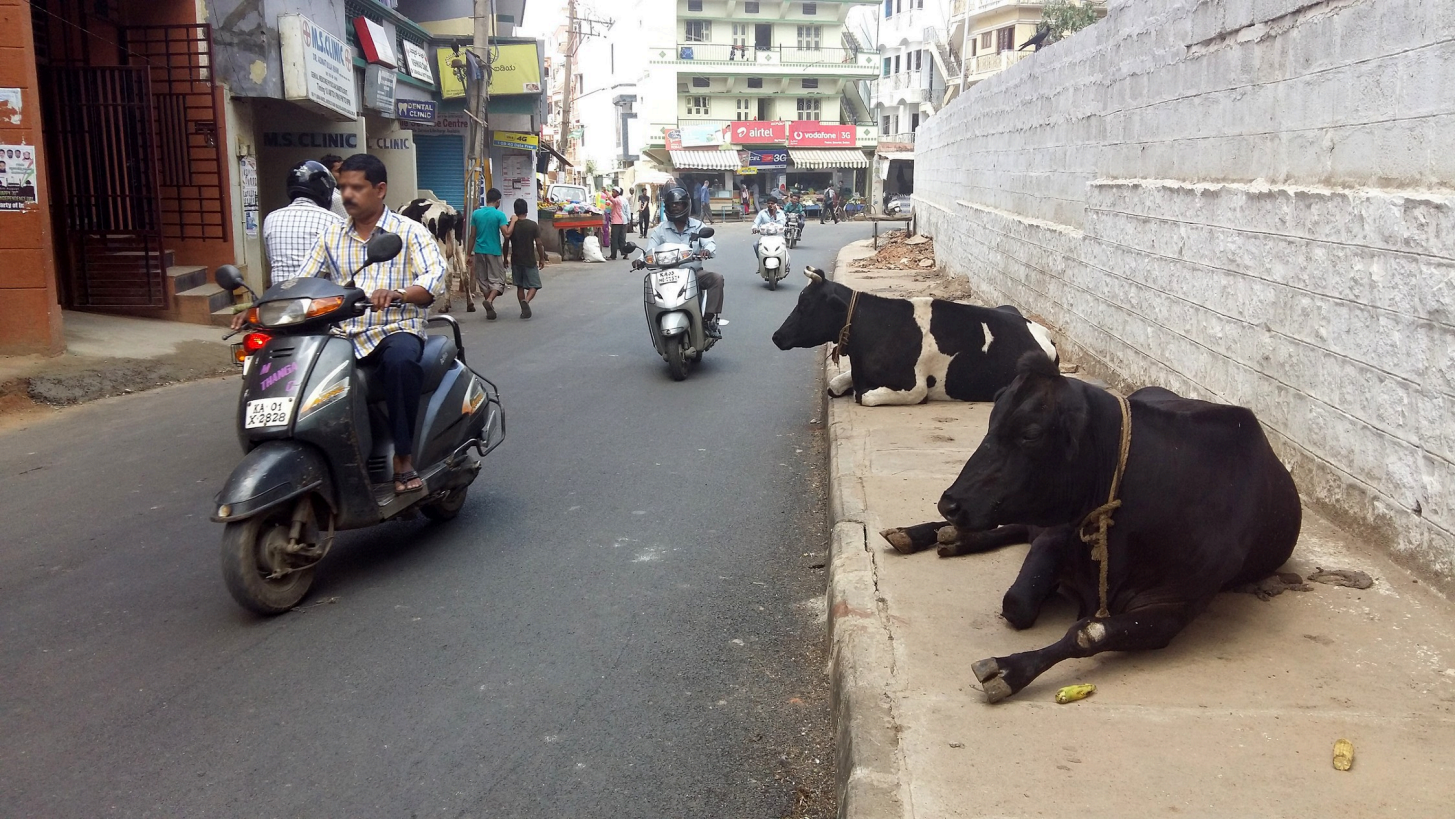
Pictures of dairy cattle in the streets and grazing on green public grounds. All pictures were taken in urban neighborhoods (SSI = 1 or 2).

**Fig 5:**
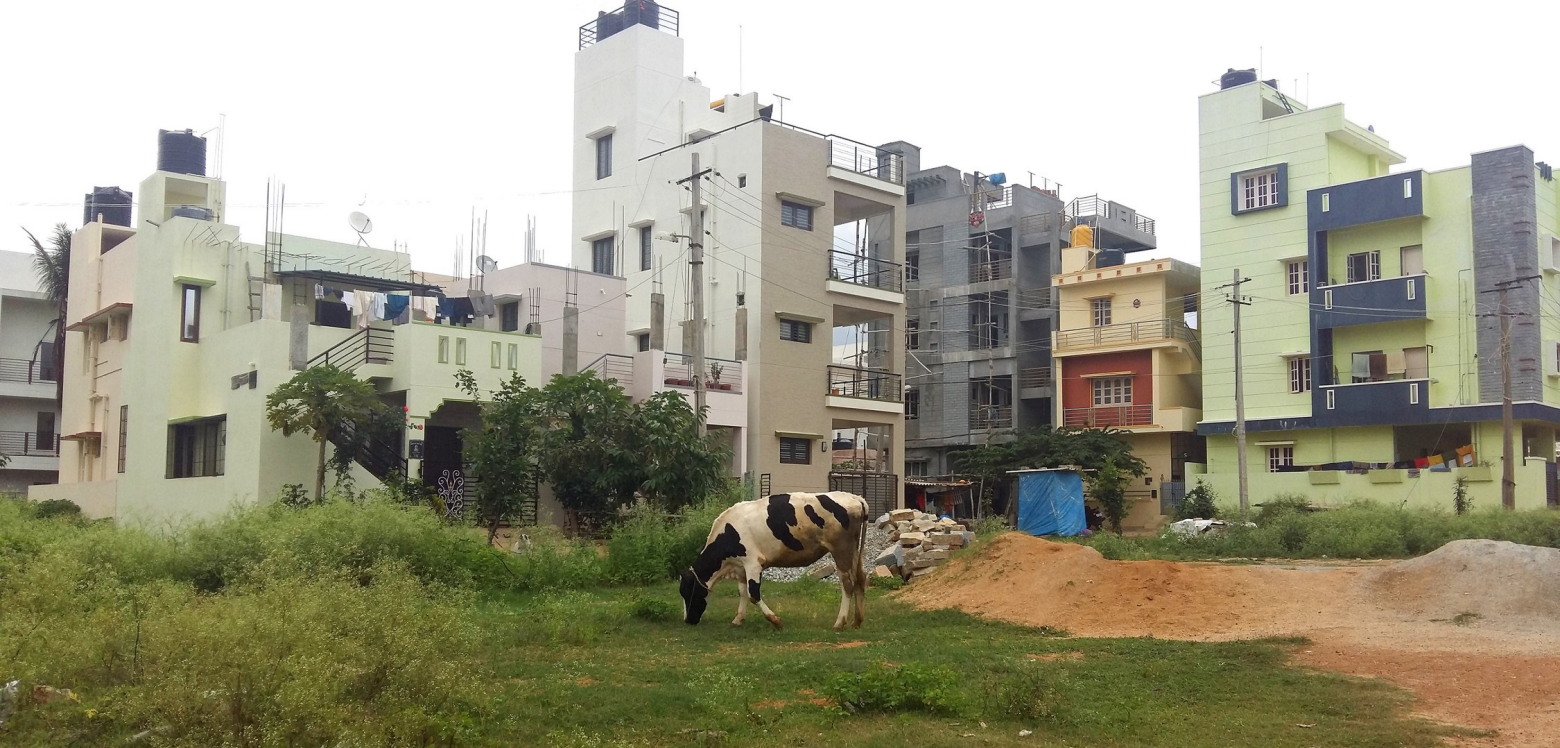
Pictures of dairy cattle in the streets and grazing on green public grounds. All pictures were taken in urban neighborhoods (SSI = 1 or 2).

**Fig 6:**
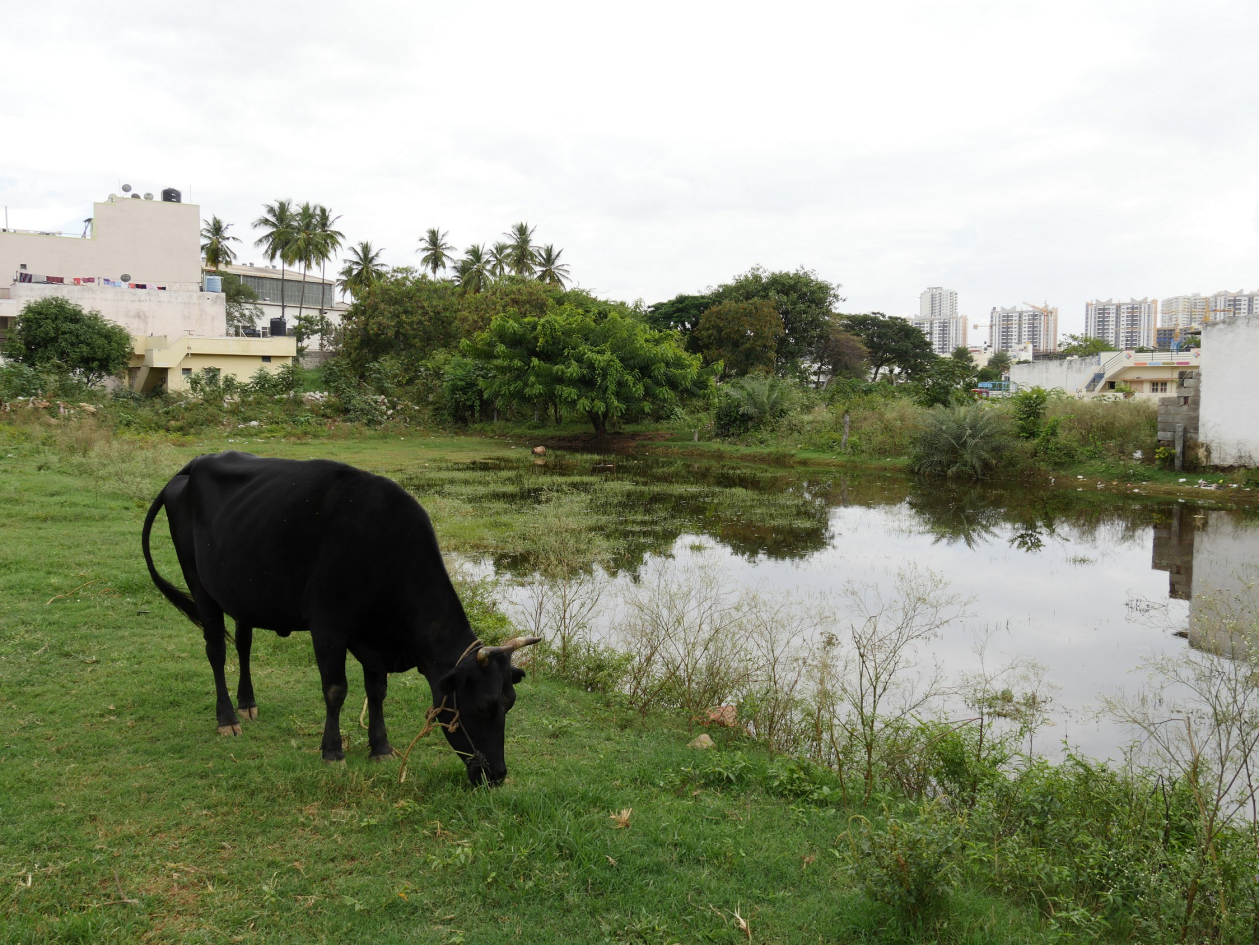
Pictures of dairy cattle in the streets and grazing on green public grounds. All pictures were taken in urban neighborhoods (SSI = 1 or 2).

As captured by P-PAS, overall, 3 dairy producers out of 4 made use of pasture: typically, the whole dairy herd, apart from the calves, was sent to pasture once per day. In line with their extensive feed management, pasturing lasted the longest with 6.6 ± 1.6 hours per day in Ext_DPS_ (P < 0.05), mainly on public grounds (80%; P < 0.05), which in urban areas include both foraging by the cows in the streets and pasture on green public grounds such as vacant plots or lakes’ surroundings (Figs 4–6). In comparison, pasturing lasted 5.9 ± 1.3 hours in Semi-A_DPS_ and 5.5 ± 1.5 in Semi-B_DPS_ and the type of pasture used was more diverse: public grounds (49% in Semi-A_DPS_; 33% in Semi-B_DPS_), public grounds in addition to their own pasture land (28% in Semi-A_DPS_; 37% in Semi-B_DPS_) or exclusively on their own pasture land (20% in Semi-A_DPS_; 30% in Semi-B_DPS_). Used of shared pasture (private pasture land belonging to family or neighbor, and used for free) occurred mainly in Ext_DPS_ (9%; P < 0.05) but rarely in Semi-A_DPS_ (3%) and not at all in Semi-B_DPS_ (0%).

**Fig 7:**
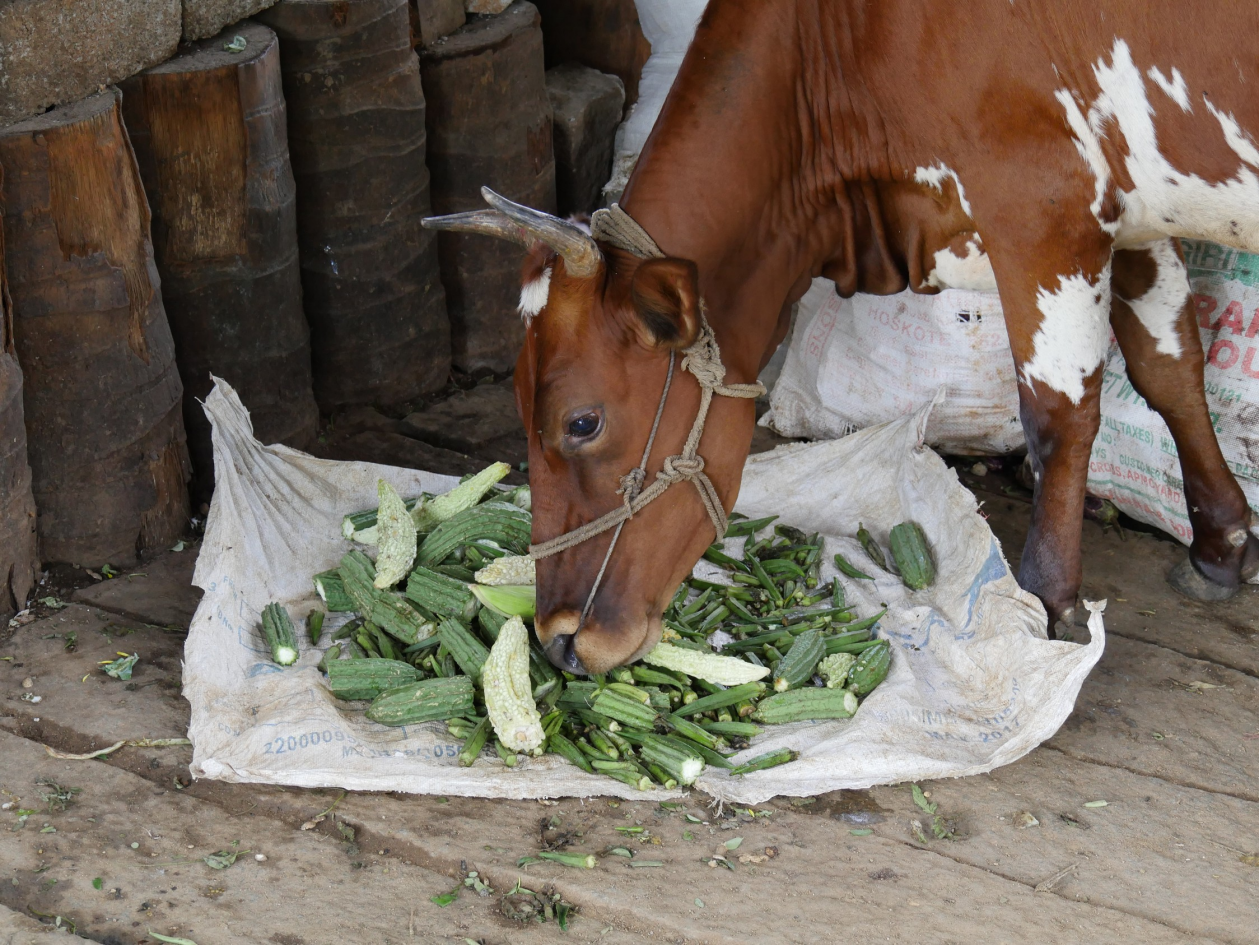
Picture of a dairy cow feeding on organic wastes collected from a vegetable market.

Nevertheless, no dairy producer relied solely on pasture and, as captured by P-FOR, 77% of them also relied, at least partially, on self-cultivated forages. Most dairy producers stated that they usually relied exclusively on their own forage production (Semi-A_DPS_ = 60%, Semi-B_DPS_ = 67%, Int_DPS_ = 68%) but 43% nonetheless had to buy forages during the last 12 months because of forage shortage. Only 9% of dairy producers had sold forage during the last 12 months. Commonly cultivated green forages were African tall maize (*Zea mays*; cultivated by 81% of dairy producers in Semi-A_DPS_, Semi-B_DPS_ and Int_DPS_) and hybrid Napier grass (hybrid *Pennisetum purpureum*; cultivated by 80%; Table 2). Although not cultivating forages, 54% of the dairy producers in Ext_DPS_ occasionally either bought (86%) or received or exchanged African tall maize through an agreement with a neighbor (14%). 56% of the dairy producers in Ext_DPS_ also either bought (79%) or received or exchanged hybrid Napier grass (21%). Because dairy producers in Ext_DPS_ partially relied on bought forages, expenses for forages were frequently mentioned as relevant in Ext_DPS_ (importance ranking = 1.5 ± 1.1; Ext_DPS_ *versus* Semi-A_DPS_, Semi-B_DPS_ and Int_DPS_; P < 0.05; Table 1). In addition to these common green forages, 39% of the dairy producers in Ext_DPS_, Semi-A_DPS_ and Semi-B_DPS_ fed their cattle with “wild grasses”: a mix of grasses naturally available in the area, collected for free on their own non-cultivated land (e.g., from field margins; depicted as “non-c wild grass” in Table 1) or on public grounds (e.g., lake shores, including lakes in urban areas; depicted as “free wild grass” in Table 1). Only 21% of the dairy producers in Int_DPS_ fed wild grasses to their cattle (P < 0.05), once more in line with their more intensive feeding management (Table 2). Across the four DPS, 83% of the dairy producers relied on straw of finger millet (*Eleusine coracanal*, known locally as *ragi*) as forage during the dry season, while rice straw feeding was uncommon (3%). As ragi is a widely cultivated staple food in the region, most dairy producers had their own ragi straw and those in Ext_DPS_ could easily and at low cost buy it from farmers. Less frequently used forages were fresh finger millet stems, sorghum (*Sorghum bicolor*) straw and, in urban areas, organic waste from fruit and vegetable markets (Fig 7). As Int_DPS_ dairy producers did not send their cattle to pasture, they fed them more often than the other dairy producers (4.6 ± 2.1 times per day *versus* 2.5 ± 1.4 in Ext_DPS_, Semi-A_DPS_ and Semi-B_DPS_; P < 0.05). Across all DPS, only 12% of the dairy producers practiced a differential feeding of forages based on physiological status of LDH. Dairy producers in Semi-A_DPS_, Semi-B_DPS_ and Int_DPS_ usually chopped forages offered to the dairy herd with a sickle (60%) while the use of a chaff cutter was rare (10% in Int_DPS_; P < 0.05). A large range of concentrate feeds was available and used by all surveyed dairy producers, either as single element or as a mixture of wheat flour, with or without bran, corn flour, dairy pellets, chickpea husks (*Cicer arietinum*, known as “Bengal gram”) and groundnut cake, to which 85% of the dairy producers added salt or a commercial mineral supplement. Concentrate feed was always bought and, although the dairy cooperatives provided concentrate feeds at affordable price, was mentioned by almost all dairy producers as their main expense, with an average importance ranking of 2.8 ± 0.8 (Table 1). Feeding household kitchen wastes to cattle was common for 86% of dairy producers in Ext_DPS_, Semi-A_DPS_ and Semi-B_DPS_, but less frequent in Int_DPS_ (69%; P < 0.05). No cattle had *ad libitum* access to water, and they were mostly offered water in the shed (82% of all herds) or in addition had access to water during pasture (river, pond, lake, 15%).

**Table 2:** Feeding frequency (in % of Dairy Producers (DP)) of the most common forages and concentrate feeds utilized in Bengaluru’s Dairy Production Systems (DPS), and, when fed by a dairy producer, their most frequent origin (own production (Own prod.), bought (Bought), collected for free from public grounds or as waste (Free) or collected on own ground but not cultivated (Non-c).

DPS	n	African tall maize		Hybrid Napier grass		Wild grass		Ragi straw		Concentrate feeds
		% of DP	Origin	% of DP	Origin	% of DP	Origin	% of DP	Origin	% of DP
Ext_DPS_	70	54*	Bought (86%*)	56	Bought (79%*)	37	Free (92%)	76	Bought (92%*)	100
Semi-A_DPS_	120	83	Own prod. (81%)	70	Own prod. (81%)	41	Free (55%)	88	Own prod. (86%*)	100
Semi-B_DPS_	76	89	Own prod. (82%)	76	Own prod. (81%)	39	Free (66%)	84	Own prod. (81%)	100
Int_DPS_	71	93	Own prod. (78%)	86	Own prod. (79%)	21*	Non-c (63%)	82	Own prod. (78%)	100

*Frequency differs significantly from overall frequency (P < 0.05).

#### Milk production and marketing

Milk production per dairy farm and day was highest in Semi-B_DPS_ and Int_DPS_ and lowest in Semi-A_DPS_ (P < 0.05), while Ext_DPS_ was in-between (Table 3). As each dairy producer kept 2.1 ± 1.3 lactating cows (only 5 dairy units had 5 to maximum 9 lactating cows at once), average daily milk production per cow made the difference, with cows in Int_DPS_ producing the highest amount of milk per day. Cows producing least were found in Ext_DPS_ and Semi-A_DPS_ (P < 0.05), while cows in Semi-B_DPS_ had an intermediary production (Table 3). Milking other than by hand, which was a time-consuming task and constrained by the dairy cooperative’s opening hours, was uncommon as only 9 dairy producers owned a milking machine. Dairy producers could not estimate the average lactation length within their herd because they usually stopped milking a cow when 7 months pregnant, irrespective of the duration of lactation. Therefore, it depended on the time a cow needed to become pregnant again and was strongly variable, as usually, more than one artificial insemination was needed. Dairy producers in Int_DPS_ preferred to feed calves with milk from a bucket (65%) instead of having it suckle the cow. In opposition, the majority of dairy producers in the other DPS allowed the calves to suckle the dam (avg. across Ext_DPS_, Semi-A_DPS_ and Semi-B_DPS_ = 62%; P < 0.05). Weaning occurred faster in Int_DPS_ (3.6 ± 1.3 months) than in all other DPS (4.6 ± 2.6 months; P < 0.05).

**Table 3:** Daily (d) milk production per Dairy Farm (DF) and per cow, share of milk kept for household (HH) consumption and use of marketing channels by the Dairy Producers (DP).

DPS	n	DF production	Cow production (liter milk cow^-1^ d^-1^)	% kept for HH consumption	Milk marketing (% of DP)			
		(liter milk DF^-1^ d^-1^)			No milk sold	Dairy coop.	Informal	Mixed
Ext_DPS_	70	20.5 ± 17.9^ab^	8.2 ± 4.4^a^	8 ± 12^a^	4	59	14*	23*
Semi-A_DPS_	120	13.6 ± 10.7^a^	7.3 ± 3.9^a^	18 ± 28^b^	8*	80	2	10
Semi-B_DPS_	76	18.7 ± 12.3^b^	8.6 ± 3.7^ab^	11 ± 19^a^	1	84	3	12
Int_DPS_	71	24.5 ± 23.1^b^	9.9 ± 4.3^b^	7 ± 6^a^	1	87	1	10

Values within a column with different superscript letters differs significantly (P < 0.05).

*Frequency within a column differs significantly from overall frequency (P < 0.05).

Dairy producers usually kept 1.2 ± 0.9 liters of milk per day for their own consumption; since dairy producers in Semi-A_DPS_ had a low total daily milk production, they kept in proportion a share twice as high as dairy producers from the other DPS (P < 0.05; Table 3). Since no dairy producer owned a cold storage facility nor processed milk into dairy products to sell them, any milk not used for household consumption was sold as raw milk. With the exception of 8% of dairy producers in Semi-A_DPS_ who did not sell any of the produced milk (P < 0.05; Table 3), all other producers marketed milk either through dairy cooperatives linked to the Karnataka Milk Federation or informal (direct) marketing channels, namely middlemen (usually delivering in bulk to restaurants) or directly to the consumer (Table 3). Across the rural clusters Semi-A_DPS_, Semi-B_DPS_ and Int_DPS_, 83% of all dairy producers delivered their milk only to their dairy cooperative; 10% delivered to their dairy cooperative and sold some liters directly to their neighbors. In Ext_DPS_, only 59% of dairy producers delivered exclusively to a dairy cooperative; since many dairy producers were located in urban and peri-urban areas, they had easier access to a larger number of consumers: 14% sold their whole milk production through informal marketing channel(s) (P < 0.05), and 23% sold part of the milk informally and relied on a cooperative for the remaining milk (P < 0.05). The dairy cooperative network was very dense: for 95% of all dairy producers who delivered milk to a dairy cooperative, the collection center was located within the same settlement as their farm. In addition to being able to easily deliver any quantity of milk twice daily on foot, dairy producers benefited from a fixed subsidy of four Indian rupees (INR; 0.05 Euro, at the time of the survey) per liter of milk. The average milk price was 23 ± 1 INR per liter (0.31 Euro), indexed on the milk’s fat content. Informal milk marketing to middlemen and consumers yielded 31 ± 5 INR per liter (0.42 Euro). The higher price and the access to consumers were the main drivers for direct milk marketing, while only four of the informal sellers claimed that they had no access to a dairy cooperative.

#### Other livestock and agricultural activities

Ownership of cattle exclusively for draught purpose in addition to dairy cattle was uncommon (8% of all dairy producers) and ownership of buffalo was rare (3%). Livestock other than cattle was encountered on 50% of the surveyed dairy farms, namely sheep and goats (raised for meat) as well as chickens (kept for eggs and meat). The number of additional livestock kept was however low, accounting for only 0.22 ± 0.74 tropical livestock units owned per household out of 3.35 ± 1.93 owned per household in total. This additional livestock was often exclusively kept for household consumption (46%) or for both household consumption and sale (37%) but seldom exclusively for sale (17%). Next to dairy production, the size of land cultivated for crops or forages, or both, averaged 1.03 ± 1.35 hectares. In 91% of the cases, the cultivated land belonged to the dairy producer. Only 2% of all dairy producers rented additional areas and 7% cultivated additional land they did not own, sometimes in exchange for a part of the crops’ or forages’ yield, corroborated by a low importance ranking (0 ± 0.1) of land as expense related to dairy production (Table 1). 84% of dairy producers in Semi-A_DPS_, Semi-B_DPS_ and Int_DPS_ pursued an agricultural activity. In Ext_DPS_, only 13% of dairy producers pursued an agricultural activity next to dairy production, cultivating crops but no forages for their cattle (P < 0.05). Generally, one dairy producer out of two was producing crops only for own household consumption. Only dairy producers in Int_DPS_ were more commercially oriented, with 23% cultivating crop solely for selling (P < 0.05), 39% for selling in addition to household consumption and only 38% exclusively for household consumption. On average 1.6 ± 1.0 crops were cultivated, ranging from finger millet and all kinds of vegetables, fruits and flowers to mulberry for sericulture. All dairy producers pursuing an agricultural activity also used their cattle manure, stored on a dung heap or in a pit, as organic fertilizer for their fields. Since the majority of dairy producers in Ext_DPS_ did not cultivate land, 65% sold their manure (P < 0.05) and 13% gave it away for free, exchanged or discarded it (P < 0.05), with the remaining 9% mentioning several uses. While manure management in rural and peri-urban areas was homogenous across DPS, alternative manure management options were encountered in urban areas, where space for dung heaps was lacking: some urban dairy producers stored fresh manure only a few days or produced dry dung cakes before selling, giving away or exchanging them. If manure was discarded, it was washed to the sewer system.

## Discussion

The analysis of dairy production within the rural-urban interface of the emerging megacity of Bengaluru provided interesting insights on the diversity of small-scale dairy production systems that supply a growing population of several million milk consumers, on their spatial distribution and on potential linkages between SES components along rural to urban gradients.

### Bengaluru’s dairy sector

A first relevant point to discuss is Bengaluru’s dairy sector, especially its overall homogeneity and its successful network of dairy cooperatives. In contrast to the immense scale of Bengaluru as a city, its dairy sector relies on numerous small-scale family businesses with a homogenous socio-economic profile. In India, 80% of dairy animals are kept in herds of 2 to 5 cows [21], a range in which Bengaluru’s average number of LDH per household (3.0 ± 1.5) fitted. Cattle and livestock ownership per household was however lower than in urban and peri-urban areas of West Africa, another urbanization hotspot of the Global South [14, 16]. While higher numbers of tropical livestock units were reported for peri-urban dairy households than for urban ones in Bobo Dioulasso, Burkina Faso [15], urban dairy producers from Bengaluru owned on the opposite more tropical livestock units than their peri-urban or rural counterparts. The average daily milk production per cow of 8.3 liters was above the average of 5.9 liters of milk per day reported for the district of Bengaluru Urban [26] but similar to milk yields of exotic crossbreeds in a typical four-dairy-animal farm in Haryana state, northern India (7.5 liters of milk per day) [30] or achieved in other urban or peri-urban dairy farms (6.7 to 11.0 liters of milk per day, Ouagadougou, Burkina Faso) [31]. Another Indian dairy production characteristic is the reliance on family labor [21, 30] as seen in Bengaluru. However, reliance on family labor can slow down intensification of labor-intensive dairy production if availability of family labor is low [32]. An interesting dynamic documented in other urban and peri-urban areas but not observed in Bengaluru is the replacement of family labor with hired labor for dairy production paid by off-farm monetary activities done by the family labor [33, 34]. New job opportunities available in the city [35], especially for a younger better-educated generation [11], might partly explain the lower number of dairy producers in urban areas as seen in Bengaluru; this deserves more research, especially since farm persistence in and adaptations to an urbanizing environment are linked to internal family dynamics [36, 37]. Similarity in number of cattle owned and reliance on family labor was reflected in the homogenous socio-economic profile of the dairy producers. The socio-economic classification of most of Bengaluru’s dairy producers as Indian middle class, to which off-farm income certainly contributed, certified their good economic situation at the country scale as the utilised Market Research Society of India’s system serves national comparison [28]. It might however not realistically reflect the dairy producers’ economic power in comparison to other inhabitants of Bengaluru as consumption inequality is generally more pronounced in urban areas [38]. Overall, Bengaluru’s dairy sector is not only homogenous for most of the production practices–herd management, breeding, health care—but also well-establish, thanks to its successful network of dairy cooperatives linked to Karnataka Milk Federation: a milk collection center existed in nearly all urban, peri-urban and rural settlements, and provided dairy producers with inputs such as exotic genotypes through artificial insemination, health check-ups, vaccinations, concentrate feeds, and extension services to improve their production. Despite being one of the largest milk producing states, 21% of the milk produced in Bihar is marketed informally [39]. In contrast, for 95% of all dairy producers across Bengaluru’s rural-urban interface, the dairy cooperatives served as the marketing channel for all or a part of their milk production, thereby fulfilling their role in i) scaling-up milk collection, processing and marketing to urban areas [21]; ii) being accessible to smallholder dairy producers, whereas private dairy processors prefer partnership with resource-rich dairy producers [40]. Through its dairy cooperatives, Bengaluru thus indeed nurtured the intensification of its dairy sector by easing access to new production inputs [12, 33].

### Bengaluru’s dairy production systems

A second relevant point to discuss is the existence of distinct DPS coexisting in Bengaluru’s rural-urban interface and the predictors they are based on. In the context of urbanization, the consideration of rural areas as a level of urbanization shifted the focus from livestock production systems in urban and peri-urban areas [14–16] to livestock production systems in an urbanizing environment. Additionally, the consideration of urbanization level as a predictor highlighted the spatial distribution of DPS across Bengaluru’s rural-urban interface but also the relative importance of location in shaping them. On one hand, the ubiquitousness of Ext_DPS_ demonstrated that a specific set of constraints *versus* opportunities in resource availability for dairy producers, namely the lack of land for the cultivation of forages *versus* the utilization of public grounds for pasture or forages collection or both, existed across urbanization levels. Such reliance of urban dairy producers on public lands or organic market wastes, or both is known in India [10] but also documented e.g. in Bobo Dioulasso, Burkina Faso [15]. Interestingly, the ubiquitousness of Ext_DPS_ showed that reliance on public lands was an extensification strategy pursued also by rural dairy producers, which could be linked to issues of land accessibility [41] or availability of family labor [11, 33–36]. On the other hand, three DPS coexisted in rural areas, highlighting a diversity of production strategies and specific sets of constraints *versus* opportunities in resource availability or environmental conditions for dairy producers even at the same urbanization level: e.g. anecdotic data suggested that the northern transect was drier than the southern one, potentially leading to a reluctance of some dairy producers in the northern transect to send their cattle to pasture because of reduced biomass availability or higher risk of heat stress due to the warmer environment, or both. In contrast, many dairy producers in the southern transect took advantage of the higher water availability by cultivating forage for their cattle, which potentially explains the higher prevalence of Int_DPS_ in the southern transect. Since improved animal nutrition and genetics are the most effective steps to improve–and intensify–dairy production [33, 42], predictors related to nutrition and breeding/herd management allowed assessing the intensification level of the four DPS. Differences in intensification level were strongest in peri-urban and rural areas, with dairy producers classified from extensive to intensive, while the majority of urban dairy producers were extensive. Concerning animal nutrition, reliance on external inputs such as buying of forage or high use of concentrate feeds is commonly seen as a step towards intensification [32] but in the context of urbanization, also as a consequence of decoupling crop and livestock production. For example, dairy producers in Cairo, Egypt, or Jimma, Ethiopia, increase the share of dry forages or concentrates feeds, or both, as a result of land scarcity, and thus feed scarcity, in their urbanizing environment [9, 43]. This practice was however uncommon in Bengaluru’s urban areas, where most of the extensive dairy producers relied on use of pasture, collected forages from public grounds or organic wastes from markets or combined all of these approaches to complement their cattle’s feed intake at the homestead, but only irregularly bought low amounts of green or dry forage. Although the results (Table 2) seem to suggest that dairy producers in Ext_DPS_ relied more on external inputs than those in the three other DPS, the share of producers purchasing green forages does neither reflect the amount of forages bought nor the regularity of purchase [44, under review]. In addition to the variables determining the distinction of the four DPS, a major factor further discriminating them was the use of concentrate feed: although purchased and fed by all dairy producers, cows’ consumption of concentrate feeds on average accounted for much less of their daily dry mater intake in Ext_DPS_ compared to Int_DPS,_ whereas it was intermediate in the two semi-intensive DPS, which furthermore did not rely on feeding organic wastes or forages from public grounds [44, under review]. Dairy producers in Ext_DPS_ thus distinguished themselves paradoxically by share of external inputs in their feeding strategy but also by their resourcefulness in relying on public grounds for forages collection and pasture, and organic wastes, thus minimizing their land, labor and financial inputs in comparison to dairy producers in Int_DPS_, who cultivated most of the green forages they offered to their dairy cattle and did not rely on pasture. This resourcefulness also applies to sourcing green forages for their dairy cattle as it was often offered as a “bonus feed” when available easily or cheaply, or both, rather than a staple of the cow’s diet.

### Social-ecological linkages within Bengaluru’s dairy production systems

A third important point to discuss are the potential linkages between producers and consumers in Bengaluru and the quality of their relationships. The main linkage between producers and consumers is the exchange of milk as a material flow against a financial one. Urbanization level however impacted producer-consumer linkages as milk flowed from peri-urban and rural producers towards Bengaluru’s urban and peri-urban consumers through the intermediary of Karnataka Milk Federation [4, 7, 8]. Vertical and horizontal integration of Bengaluru’s formal dairy value chain was strong, as Karnataka Milk Federation dominated all processes from rural milk collection to urban distribution of dairy products. Producer-consumer linkages in urban areas were diverse, ranging from informal direct customer linkage (neighbors) to informal indirect (restaurant through middleman) and formal indirect ones (dairy cooperatives), which reflects the general diversity of India’s dairy sector [10, 21, 37]. As in Nakuru, Kenya [34], informal urban channels in Bengaluru were financially more rewarding. Producer-consumer linkages in urban areas are also supported by the consumers’ preference for fresh raw milk over processed milk, awareness of health risks of raw milk—thus boiling freshly sourced milk before consumption—and higher trust in a direct producer-consumer linkage [45]. At last, producer-consumer linkages are supported by the socio-cultural services provided by cows: as a holy animal, their presence is enjoyed and they are still part of many religious ceremonies, such as blessing of a new house [46, 47]. Urban milk collection points of the cooperatives were less easily accessible than rural ones (far distance between farm and collection point, therefore not accessible by foot) and served as backup to sell milk leftovers, while the access to provided inputs (artificial insemination, veterinary care, concentrate feeds) was more variable. Urban dairy production in Bengaluru thus not only provides fresh milk directly to consumers but also an opportunity for dairy producers to continue their economic activity in a city that literally grew around them, while integrating themselves into the urban landscape, benefitting from improved infrastructure (schools, hospitals) and preserving their cultural identity [6]. Cattle are however paying the price of this urban integration as they are not well-adapted to urban husbandry conditions [10], and are at risk of ingesting plastic waste on the many uncontrolled waste dumps when foraging in the streets [46, 48]. The most important difference between rural and urban SES linkages at farm-level related to manure handling and the decoupling of crop and livestock production in urban areas. Not only did Bengaluru act as a nutrient sink [49] but the manure was sometimes washed away to avoid neighbors’ complaints about bad odor and flies [7, 10], potentially polluting Bengaluru’s water bodies [10]. At landscape-level, the extensive strategy of urban dairy producers thus trades off a social benefit, i.e. the integration of dairy producers within Bengaluru, for a negative externality, i.e. manure mismanagement, and poor husbandry conditions [50].

## Conclusions

The case study of dairy production in the urbanizing environment of Bengaluru’s rural-urban interface demonstrates that distinct dairy production systems coexist along a rural-urban gradient. Addressing the urbanization level as a clustering variable reveals spatially explicit trends of intensification as well as social-ecological linkages. Despite rapidly progressing urbanization and a population of 10 million, Bengaluru’s dairy sector relies on small-scale family dairy farms and a strong network of dairy cooperatives connecting dairy producers in remote rural settlements to the urban consumers, thereby sustaining dairy production and livelihood of the producers. Distinct feeding and breeding/herd management practices result in several intensification levels across Bengaluru’s rural-urban interface. Changes in resources availability, such as land and labor, are potential drivers of market-oriented intensification but also of extensification of dairy production in an urbanizing environment. The megacity of Bengaluru represents an especially challenging and highly land competitive environment, which puts at question the long-term viability of urban and peri-urban dairy production and its role for satisfying the demand for milk of a growing population of urban consumers.

## Supporting information

S1 Table Number of selected settlements and completed dairy production baseline surveys in the northern research transect (Nsect), in the southern research transect (Ssect) and in additional locations (Add.) per survey stratification index (SSI) stratum and urbanization level. (PDF)
