# Supplementary material for: Dairy production in an urbanizing environment—Typology and linkages in the megacity of Bengaluru, India
Source: PLoS One. 2021 Aug 12;16(8):e0255791. doi: 10.1371/journal.pone.0255791 (PMC8360525; doi:10.1371/journal.pone.0255791)
Supplement: S1 Table — (PDF) [file pone.0255791.s001.pdf]

S1 Table. Number of selected settlements and completed dairy production baseline surveys in the northern research transect (Nsect), in the southern research transect (Ssect) and in additional locations (Add.) per survey stratification index (SSI) stratum and urbanization level.

Urbanization level	SSI	**Settlements (n)**				**Surveys (n)**				
		Nsect	Ssect	Add.	Total	Nsect[†]	Ssect[‡]	Add.	Total	Percentage
Urban	Stratum 1	1	2	1	4	3	8	4[‡]	15	9%
	Stratum 2	1	2	1	4	8	5	2[†]	15	
Peri-Urban	Stratum 3	1	2		3	13	13		26	33%
	Stratum 4	3	4		7	38	46		84	
Rural	Stratum 5	5	4		9	43	87		130	58%
	Stratum 6	1	1		5	45	22		67	
	Overall	15	15	2	32	150	181	6	337	

[†]selection threshold per settlement of 20%

[‡]selection threshold per settlement of 30%
